# The Role of Brassinosteroids in Controlling Plant Height in *Poaceae*: A Genetic Perspective

**DOI:** 10.3390/ijms21041191

**Published:** 2020-02-11

**Authors:** Giulia Castorina, Gabriella Consonni

**Affiliations:** Department of Agricultural and Environmental Sciences—Production, Landscape, Agroenergy (DISAA), Università degli Studi di Milano, Via Celoria 2, 20133 Milan, Italy; giulia.castorina@unimi.it

**Keywords:** brassinosteroids, cell elongation, gibberellins, *Hordeum vulgare*, *Oryza sativa*, plant development, plant mutants, plant stature, transcriptional regulation, *Zea mays*

## Abstract

The most consistent phenotype of the brassinosteroid (BR)-related mutants is the dwarf habit. This observation has been reported in every species in which BR action has been studied through a mutational approach. On this basis, a significant role has been attributed to BRs in promoting plant growth. In this review, we summarize the work conducted in rice, maize, and barley for the genetic dissection of the pathway and the functional analysis of the genes involved. Similarities and differences detected in these species for the BR role in plant development are presented. BR promotes plant cell elongation through a complex signalling cascade that modulates the activities of growth-related genes and through the interaction with gibberellins (GAs), another class of important growth-promoting hormones. Evidence of BR–GA cross-talk in controlling plant height has been collected, and mechanisms of interaction have been studied in detail in *Arabidopsis thaliana* and in rice (*Oryza sativa*). The complex picture emerging from the studies has highlighted points of interaction involving both metabolic and signalling pathways. Variations in plant stature influence plant performance in terms of stability and yield. The comprehension of BR’s functional mechanisms will therefore be fundamental for future applications in plant-breeding programs.

## 1. Introduction

Brassinosteroids (BRs) are a class of plant steroid hormones, showing structural similarity to the steroid hormones of mammals. Their production involves a network of reactions forming a complex biosynthetic pathway. More than 50 different brassinosteroids have been identified in plants, including numerous intermediates and brassinolide (BL), the final product of the pathway, which is generated by conversion from castasterone (CS) [1,2].

BR action is essential for plant growth and development. They modulate gene expression and control a vast range of processes including cell division and elongation, plant growth, vascular differentiation, and reproductive development [3]. They are also involved in developmental processes such as seed germination, leaf angle, flowering time, and seed yield, which are of great agronomic importance [4,5]. In addition, BRs play an important role in conferring tolerance to biotic [6] and abiotic [7,8] stress conditions. For all these reasons, this class of hormones has received much attention since their discovery 40 years ago, and much progress has been made in understanding the molecular mechanisms involved in BR metabolism as well as perception and signalling.

Important achievements in understanding BR biosynthesis and action have been obtained by means of forward genetic approaches in which the phenotypes of BR-deficient mutants were carefully described and provided important tools for the identification of metabolic and signalling components and revealing the mechanisms at the basis of the interaction between BRs and other plant hormones. Extensive analyses have been performed in the model plant species *Arabidopsis thaliana*. Nine genes have been described so far that code for enzymes participating in the biosynthetic pathway, from episterol to brassinolide. Several of these enzymes have broad substrate specificity and catalyse multiple steps of the pathway [5].

In this review, we focus on the genetic and molecular analyses carried out in important cereal species with the aim of unravelling the role and mechanisms of action of this class of hormones. Studies performed in maize (*Zea mays*), barley (*Hordeum vulgare*), and rice (*Oryza sativa*) are reported. The genetic dissection of the metabolic and signalling pathways have been mainly achieved in these species through forward genetic analysis. In barley, the analysis of cultivated genetic material also provides interesting tools. Lack of BR action causes an evident defect in plant stature. On this basis, a significant role has been attributed to BR in promoting plant growth in model as well as crop species. Besides Arabidopsis, which, as already mentioned, has been referred to as an important model for dissecting BR mode of action, mechanisms underlying BR-mediated plant growth have so far been characterized in great detail in rice. In this species, interesting achievements in the comprehension of BR action as well as of the mechanisms of interaction between BR and gibberellin (GA) have been obtained and are reported in this work with the aim of describing the most significant components. Overall, these studies have highlighted a complex picture in which different mechanisms and effects exist, depending on the tissue and developmental phases, and are strongly related to hormone concentration.

## 2. Dissection of BR Biosynthesis in Maize, Rice, and Barley through Mutant Analysis

**Figure 1:**
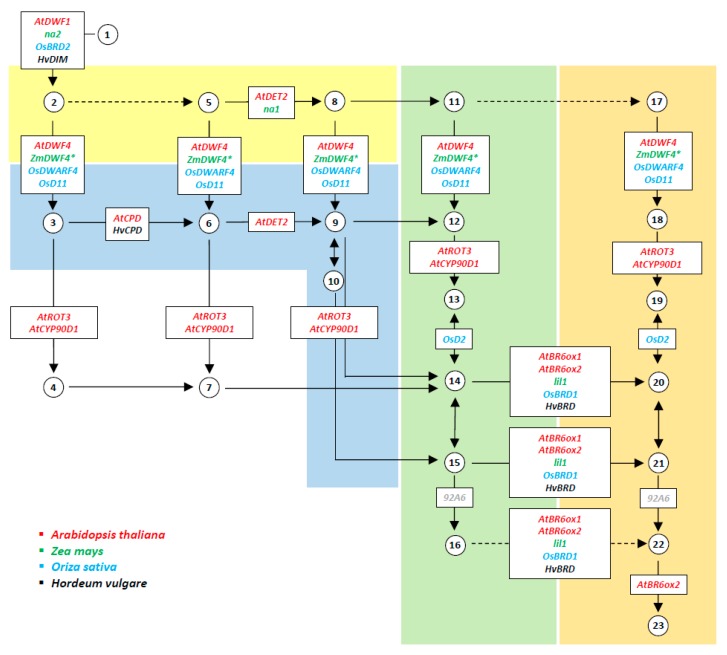
Simplified brassinosteroid (BR) biosynthetic pathway. Late C-22 oxidation, early C-22 oxidation, late C-6 oxidation, and early C-6 oxidation pathway are highlighted in yellow, blue, green, and orange, respectively. The symbols of genes encoding enzymes are represented by the arrows indicating specific reaction steps. Arrows without gene symbol are referred to by steps with uncharacterized enzymes. Dashed arrows indicate multi-enzymatic steps. The second-to-last enzymatic step of the pathway is catalysed by a putative typhasterol/6-deoxotyphasterol 2alpha-hydroxylase (92A6) that has yet not been characterized in cereals. The BR intermediates are shown as numbers within circles as follows: (1) 24-methylenecholesterol; (2) campesterol; (3) 22alpha-hydroxy-campesterol; (4) (22R,23R)-22,23-dihydroxycampesterol; (5) campest-4-en-3-one; (6) 22alpha-hydroxy-campest-4-en-3-one; (7) (22R,23R)-22,23-dihydroxy-campest-4-en-3-one; (8) 5alpha-campestan-3-one; (9) 22alpha-hydroxy-5alpha-campestan-3-one; (10) 3-epi-6-deoxocathasterone; (11) 5alpha-campestanol; (12) 6-deoxocathasterone; (13) 6-deoxoteasterone; (14) 3-dehydro-6-deoxoteasterone; (15) 6-deoxotyphasterol; (16) 6-deoxocastasterone; (17) 6-oxocampestanol; (18) cathasterone; (19) teasterone; (20) 3-dehydroteasterone; (21) typhasterol; (22) castasterone, and (23) brassinolide. (Modified after the Brassinosteroid biosynthesis—Reference pathway available at KEGG; https://www.genome.jp/kegg-bin/show_pathway?map00905).

BR-related mutants have been characterized in maize, rice, and barley and allowed the isolation of genes involved in the biosynthetic pathway. As illustrated in Figure 1, each gene has been assigned to one or more specific biosynthetic steps. In addition, correspondence among genes of the different species has been established according to sequence features and their function (Table 1). In rice (*Oryza sativa*), seven genes have been identified, and are hereafter reported, which are involved in the BR metabolic pathway. Mutants in these genes cause a pleiotropic phenotype (Table 2). The most evident effect consists in reduced height (between 20% and 30% less than the wild type), but reduced leaf length and shortened grains have also been observed. An additional distinct feature observed in these mutants consists of the presence of erect leaves that differ from wild type leaves, which bend away from the vertical axis. Both leaf dwarfing and/or the bending angle of the lamina joint can be restored by BL treatment. Reduced plant height is due to reduced internode elongation. At the tissue level, it has been shown that the reduction in the elongation of both leaf blades and sheaths is due to lack of cell polar elongation [9,10]. BR effect on plant height is mediated by the control exerted by these hormones on shoot apical meristem development, cell division, and microtubule formation and orientation [9,10,11]. The effect on microtubule formation had already been investigated in Arabidopsis. The study of the *bul1-1* (*boule 1-1*) dwarf mutant, lately renamed *dwarf 7*, defective in the BR pathway revealed that very few microtubules were present in the elongation zone and that the parallel microtubule organization, which is typical of wild type elongating cells, was lacking. However, BL treatment could rescue this mutant cellular phenotype [12].

Starting from upstream, *brassinosteroid-deficient dwarf2* (*OsBRD2*) is the first characterized rice gene in the biosynthetic pathway. Its identification and functional characterization, through the analysis of the *DIMINUTO/DWARF1 DIM/DWF1* mutant, showed that the *brd2* gene encodes for a C-24 sterol reductase that promotes, in an initial step of the pathway, the conversion of 24-methylene-cholesterol (24-MC) to campesterol (CR) [13]. Differently from the other mutants affecting enzymatic steps located more downstream in the pathway, which exhibit an extreme reduction in plant height, *brd2* shows a moderate phenotype, particularly evident at an early vegetative stage. The detection in these mutant lines of trace levels of castasterone (CS) provided an explanation for the attenuated the severity of the phenotype. This observation also pointed to the existence of an alternative BR biosynthetic pathway that leads to the production of the active BR molecules.

The genetic molecular analysis of the rice *ebisu dwarf* mutant led to the identification of the *D2* gene (*OsD2*) encoding a cytochrome P450 enzyme, classified as CYP90D2, which catalyses the C-3 oxidation steps [14], whereas the analysis of the rice *dwarf11* (*d11*) mutant revealed a defect in the *OsD11*/*OsDwarf4L1*/*OsCPB1* gene encoding a C-22α hydroxylase (CYP724B1), which was suggested as being involved in the supply of 6-deoxotyphasterol (6-deoxoTY) and typhasterol (TY) [15]. The rice *d11* and *osdwarf4-1* mutants showed typical traits including erect leaves in the mature stages, shortening of the second internode in the culm, and reduced grain length. In rice, C-22 hydroxylation is also controlled by the CYP90B2/OsDWARF4 paralog [16]. Differently, a single gene—*Zmdwarf4—*encoding 22α-hydroxylase has been found in maize [17]. Redundancy was also observed in rice for C-23 hydroxylation that was shown to be controlled by CYP90A3/OsCPD1 and CYP90A4/OsCPD2 [18].

The final steps of the pathway are controlled by *brassinosteroid*-*dependent 1* (*OsBRD1*) encoding a BR-6-oxidase. Lack of this enzyme in the corresponding mutant causes a phenotype very similar to that observed for the other mutants in biosynthetic genes and consists of a drastic reduction in the elongation of both leaf blades and sheaths [9,10]. In addition, *brd1* mutant showed curled and frizzled leaf blades and produced only a few small and sterile seeds [10].

The first identified BR-related mutant in rice was indeed a dwarf mutant named *d61*, which is defective in the rice *brassinosteroid insensitive-1* (*Bri1*) gene encoding for OsBRI1, the BR receptor kinase [11]. As in Arabidopsis, the gene is expressed in almost all organs [11]. This mutant and the additional loss-of-function mutants *d61-1* and *d61-2* displayed similar phenotypes as biosynthetic mutants, including erect leaves, dwarf culms, abnormal skotomorphogenesis, and no organized microtubule arrangement in the cells from non-elongated internodes. Interestingly, the detailed analysis of the most severe mutant allele, *d61-4*, showed that developmental processes such as pattern formation and differentiation were normal. This suggests that BR action is not required for organ initiation and morphogenetic processes but is necessary for controlling cell division and elongation during organ development. The severe shoot phenotype observed in BR mutants was therefore caused by a defect in cell elongation and the disturbance of cell division that occurred after the determination of cell fate [19]. Besides *Bri1*, several additional genetic components of the signalling pathway have been analysed in rice, which are mentioned in the following section.

A forward genetic approach was mostly undertaken in maize (*Zea mays*) to dissect the BR biosynthetic pathway, and mutants so far described in this species are ascribable to three genes encoding biosynthetic enzymes. The *nana plant2* (*na2*) gene, corresponding to *brd2* in *A. thaliana*, encodes a C-24 sterol reductase, which is responsible for the reduction of 24-methylenecholesterol to campesterol [20,21]. The *nana plant1* (*na1*) gene, which has been shown to be the homolog of the *A. thaliana* gene *DEETIOLATED2* (*DET2)*, encodes a 5α-reductase enzyme, catalysing the 5α-reduction of (24R)-24-methylcholest-4-en-3-one to (24R)-24-methyl-5α-cholestan-3-one [22,23]. The final steps of the biosynthesis are controlled by the *Zmbrd1/lil1* (*brassinosteroid-deficient dwarf1*/*lilliputian1*) gene [24,25], encoding a BR C-6 oxidase that belongs to the large family of cytochrome P450 [26].

It should be noted that in maize, as well as in rice, one CYP85A gene exists, which is involved in the final steps of the BR pathway. Two genes were instead detected in the Arabidopsis (*Arabidopsis thaliana*) and tomato (*Lycopersicon esculentum*) genomes, most probably arising from a duplication that occurred in the genome of dicots. One of the two paralogs is more specifically involved in controlling plant growth, whereas the other one seems involved either in female gametogenesis [27] or fruit development [28].

It has also been shown that the rice C-6 oxidase functions as CS but not BL synthase and that rice plants lack an endogenous pool of BL [9,11,13,19,29]. Altogether, these results suggest that rice plants use CS as a bioactive BR compound instead of BL, an observation that might be extended to all monocot species so far analysed.

Maize mutants with deficiency in the above-mentioned genes of the BR pathway show a dramatic decrease in plant height when compared with wild types. Similarly to rice, the dwarf stature was attributable to a reduction of internode length and not a reduction in internode number. Dark green and less elongated leaves were also observed. The observation that the reduction in plant height is more dramatic in mutants impaired in the last steps of the pathway is also analogous to what was previously reported in rice. The phenotype of the *lil1-1* mutant, impaired in the last reaction, is more severe and is epistatic to that of the *na1*-*1* allele that controls an earlier step of the pathway. It was thus proposed that an additional branch of the pathway, besides those controlled by *na1*, leads to the production of CS precursors [25].

An additional symptomatic trait, that is, altered leaf angle, was described for the *na2-1* mutant, whose leaves were more upright than those of wild-type controls [20]. No effect on seed size and weight was instead observed in maize mutants lacking active BR molecules [25]. A distinct trait observed in maize mutants is the impairment in sex determination. Maize is a monoecious plant, in that it produces separate inflorescences carrying unisexual flowers, called florets, on the same plant. The tassel, which is produced at the tip of the uppermost plant internode, represents the male inflorescence, whereas female inflorescences, called “ears”, are formed on lateral branches. In normal plants, perfect (hermaphroditic) flowers are primarily formed in both inflorescences, and monoecy is achieved by the selective abortion of pistil primordia in flowers of the tassel and of stamen primordia in ear flowers [30]. As clearly shown in the work of Hartwig et al. [22], dwarf *na1* mutant plants showed feminized male flowers, which consist of mutant tassels presenting pistil development. This indicates that in maize, BR hormones are involved in promoting the abortion of pistil primordia, which normally occurs in wild type tassels during their development. The approach adopted for the functional characterization of the maize BR receptor BRASSINOSTEROID INSENSITIVE-1 (BRI1) consisted in the knockdown, via transgenic RNA interference (RNAi), of the expression of all five maize *BRI1* homologous genes [31]. The resulting mutant pleiotropic phenotype included the typical traits related to BR deficiency such as dwarf stature due to shortened internodes; dark green, upright, and twisted leaves with decreased auricle formation; and feminized male flowers.

A small group of BR-related genes were characterized in barley (*Hordeum vulgare*). The BR receptor was identified at first from the analysis of the semidwarf phenotype present in barley germplasm. This trait was attributed to an allele carrying a nucleotide substitution in the *Uzu1* gene, the barley gene orthologous to the *BRASSINOSTEROID INSENSITIVE-1* (*BRI1*) gene of Arabidopsis [32,33]. The mutant allele, named uzu1.a, carries a His857Arg modification in the kinase domain of the BR receptor [33]. Because conferring traits of agronomic importance, such as a short and sturdy culm, it has widely spread in winter barley cultivars grown in Asia.

Additional distinctive traits, including an upright plant architecture, acute leaf-blade angles, short-awned compact spikes with dense basal spikelets, irregular elongation of rachis internodes and leaf margins and auricles with undulating appearance were found in this mutant [34]. The same traits were also detected in wild type plants treated with propiconazole, a strong inhibitor of brassinosteroid biosynthesis [22]. Altogether, they constitute an ideotype of barley plants with BR deficiency, and were considered as diagnostic for the survey of a wide collection of historic barley dwarf mutants [34]. To distinguish between metabolic and signalling mutants, the detected candidates were subjected to two additional tests. Exogenous BL treatments were administered to seedlings to test for the increased leaf inclination and to dark grown leaves for the so-called “leaf-unrolling assay”. Mutants insensitive to this test were considered as candidates for signalling components.

Genetic and sequence analysis of the selected lines allowed for the identification of a mutant in the BR signalling gene (*BRASSINOSTEROID INSENSITIVE-1* (*HvBRI1*)) and in three novel biosynthetic genes. The three biosynthetic genes were assigned to different steps of the pathway. *HvBRD* encodes the BRASSINOSTEROID-6-OXIDASE catalysing the biosynthesis of castasterone, *CONSTITUTIVE PHOTOMORPHOGENIC DWARF* (*HvCPD*) encodes the C-23a-hydroxylase cytochrome P450 90A1 (CYP90A1), and *DIMINUTO* (*HvDIM*) encodes a C-24 sterol reductase [34]. Correspondence among these genes and genes in rice, maize, and Arabidopsis is reported in Table 1.

## 3. From BR Signal Perception to the Regulation of Cell Elongation Processes

The most comprehensive description of BR signalling has been achieved in Arabidopsis [35], whereas rice (*Oryza sativa*) is the only grass species in which this process has been defined in some detail. The BR signal has been shown to be perceived by the membrane localized Leu-rich repeat receptor-like kinases OsBRI1 and its coreceptor BRI1-ASSOCIATED RECEPTOR KINASE 1 (OsBAK1) [11,36].

**Figure 2:**
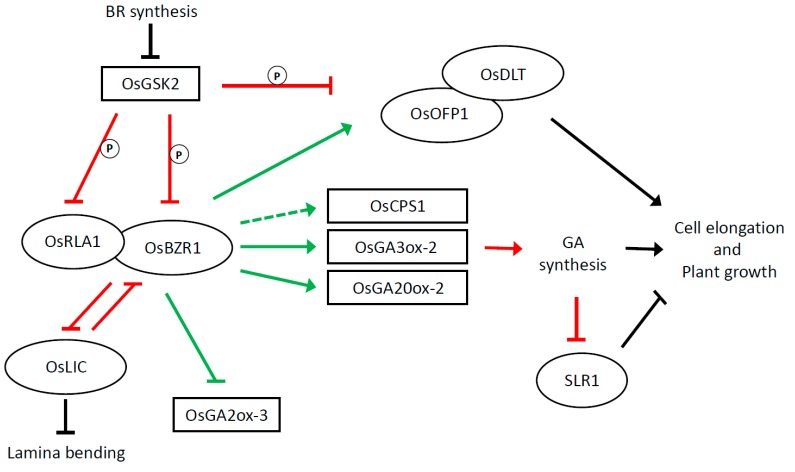
BR regulation of plant cell elongation under physiological conditions in rice. Diagram illustrates main components and type of interactions among them. GSK2 and *Os*BZR1 are two important actors in this pathway. GSK2 is a cGSK3-like kinase encoded by a rice orthologue of Arabidopsis BR INSENSITIVE 2 (BIN2); it targets and thus inhibits the functions of the OsBZR1 transcription factor [40] and a number of transcription factors (TFs) involved in BR response. Phosphorylation affects their nuclear localization and may suppress its activity. OsBZR1 targets gibberellin (GA) metabolic genes such as GA20ox-2, GA3ox-2, and GA2ox-3. Members of the GA20ox and GA3ox families are involved in the last steps of the synthesis of active GA, whereas GA2ox members are involved in inactivation of the biosynthesis. OsBZR1 exerts a positive effect on the first two and a negative effect on the third. The BR signal leads to the inactivation of GSK2, thus releasing the repression exerted by GSK2 on BZR1 [37,38,39,40]. OsBZR1 is thus “free” to promote GA synthesis and consequently plant growth. Promotion of GA synthesis causes the degradation of SLR1, a DELLA protein that negatively regulates GA signalling. This enhances the BR-promoting effect on plant growth. Unphosphorylated REDUCED LEAF ANGLE 1 (RLA1) interacts with OsBZR1 and enhances its transcriptional activity. DWARF AND LOW-TILLERING (DLT), a GRAS family protein, was shown to interact with OFP1 (ovate family protein 1). A dual regulation is exerted on OFP1. Its activity is enhanced at the protein level as a consequence of the GSK2 inactivation and at the transcription level because OsBZR1 binds to its promoter and induces its expression. DLT–OFP1 interaction positively regulates BR response [46]. *OsLIC* (LEAF and TILLER ANGLE INCREASED CONTROLLER) encodes for a C3H-type transcription factor; it interacts with and antagonizes OsBZR1 to negatively regulate BR responses [43]. It is involved in plant height as well lamina bending. Ovals represent transcription factors; rectangles indicate other proteins. Solid green and red lines indicate interaction at the transcriptional and post-transcriptional level respectively. The dotted arrow indicates that the binding at the promoter level was not shown. T lines and arrows are black if the mode of interaction is not known.

The main regulatory components of the downstream pathways are illustrated in Figure 2. An activated phosphatase dephosphorylates and represses the GSK2 kinase BR INSENSITIVE 2 (BIN2) [37,38,39,40], thus releasing the repression on OsBZR1, a transcription factor (TF) that targets and regulates different BR-responsive genes and promotes BR signalling [41]. This kinase is considered to be one of the main actors in BR signalling, as it modulates BR response through targeting and thus inhibits the functions of a number of TFs. Among them, *DWARF AND LOW-TILLERING* (DLT) [42], *LEAF AND TILLER ANGLE INCREASED CONTROLLER* (LIC) [43], *REDUCED LEAF ANGLE 1/SMALL ORGAN SIZE 1* (RLA1/SMOS1) [44], and *OVATE FAMILY PROTEIN 8* (OFP8) [45] are all involved in cell elongation.

RLA1/SMOS1 is phosphorylated by GSK2 and thus inactivated or subjected to degradation. If BR presence prevents against degradation, this protein can interact with OsBZR1 to enhance its transcriptional activity [44]. OFP1 is also considered an important positive player in the BR-mediated elongation process. The expression of this member of the ovate family proteins (OFPs) of plant-specific transcription factors [45], in physiological condition, is induced by OsBZR1, which physically interacts with the *OFP1* promoter. Moreover, OFP1 stability is enhanced by the inactivation of GSK2 triggered by the BR signal [46]. OFP8 is another a positive regulator of BR response involved in lamina bending. It was shown that, if phosphorylated, OFP8 shuttles from the nucleus to the cytoplasm where it is degraded by the proteasome [47].

Besides this network, other branches are involved in BR signalling. For example, INCREASED LAMINAR INCLINATION (ILI1) [48], and BRASSINOSTEROID UPREGULATED1 (BU1) [49] two transcription factors containing the basic HLH (bHLH) domain but lacking basic regions and standard bHLH proteins, and the OsBUL1 complex 1 (OsBC1) [50], are consistently induced by BR.

On the basis of the cellular phenotypes observed in BR-deficient mutants, downstream target genes might be genes whose products are involved in cell elongation. Organization of cortical microtubules is crucial for cell elongation. As reported in Arabidopsis, growth of hypocotyl cells requires that the parallel array of cortical microtubules is mainly transversely oriented, whereas the elongation of these cells stops if microtubules are longitudinally oriented. Although a complete picture of the molecular mechanisms involved is not yet available, there is strong evidence that the BR signal directly influences the stabilization of microtubules. BIN2 was shown to interact both in vitro and in vivo with tubulin proteins and, through its effect on microtubules, directly regulates pavement cell development and consequently organ elongation [51].

In this context, an important player was detected, namely, MICROTUBULE DESTABILIZING PROTEIN 40 (MDP40). MDP40, a positive regulator of hypocotyl cell elongation, is targeted by BZR1 and thus mediates BR regulation of cortical microtubule reorientation [52]. The functional study of OsBUL1 encoding a transcription factor with an atypical HLH protein showed that this protein interacts with LO9-177, a KxDL motif-containing protein, which acts as a mediator between BRASSINOSTEROID UPREGULATED LIKE 1 (OsBUL1) and OsBC1, a typical bHLH transcription factor that promotes lamina bending. This work provides a first picture of the mechanisms constituting the functional network at the basis of cell elongation in rice, in which a novel protein complex, consisting of OsBUL1, LO9-177 and OsBC, associated with the HLH-bHLH, is involved [50].

## 4. BR–GA Interaction in Controlling Plant Height

It has been clearly demonstrated in rice and in Arabidopsis that plant growth is regulated by the interaction between BRs and gibberellins (GAs), another class of well-known growth-promoting hormones. BR–GA interaction influences cell elongation, though it is not required to promote other processes controlled by BR, such as grain size, to which GAs contribute to a very limited extent [53,54]. Analogously, it was shown that BRs, but not GAs, are involved in skotomorphogenesis in rice [11].

The detection of the physical interaction between BZR1, the transcription factor involved in BR signalling, and DELLAs, the negative components of GA signalling, was first shown in Arabidopsis. GA induces DELLA degradation and releases BZR1 from the BZR1–DELLA complex that causes the inhibition of DNA-binding ability of BZR1, thus promoting cell elongation [55,56,57]. This was first considered as the crosstalk point between the BR and GA signalling pathways.

Strong evidence has lately shown that the interaction occurs not only at the signalling but also at the metabolic level for both rice and Arabidopsis. In rice, it was reported that GA levels were consistently decreased in mutants with impairments in BR biosynthesis. Moreover, in BR signalling mutants as well as in lines with overexpression of BR signalling components, GA levels correlated well with plant seedling height [58]. Expression analysis conducted in a group of BR-related rice mutants showed that GA levels and the expression levels of genes involved in GA metabolism, including GA20ox-2/SD1 and GA3ox-2/D18, were stimulated by BR [58]. These genes, all belonging to the cytochrome P450 oxidase gene family, are mainly expressed in seedling tissues where the main active form is GA_1_. The main target of GA stimulation promoted by BR appeared to be GA3ox-2, encoding the enzyme catalysing the change from GA_20_ to bioactive GA_1_, with its expression being found to be considerably stimulated by BR. An opposite pattern was instead detected for GA2ox-3, the GA inactivation gene, whose expression is repressed by BR. As illustrated in Figure 2, the proposed model implies that BR promotes GA biosynthesis and inhibits GA inactivation, and this leads to increased GA levels and cell elongation. It is sustained by the demonstration that the regulatory factor BZR1 directly binds to promoters of GA biosynthetic genes. Moreover, the observation that corresponding GA mutants are insensitive to BR supports the hypothesis of the interaction at the metabolic levels. This positive action exerted by BZR1 might be also assisted by other TFs involved in BR signalling, such as the above-mentioned DLT and RLA1/SMOS1. It was also observed that the effect of BR on plant growth can vary according to the hormone concentration in a certain tissue.

High levels of BRs, such as those obtained through external BL (brassinolide) application, were shown to have inhibitory effects on rice growth. BL repression was found to be mostly mediated by the upregulation of the GA inactivation gene GA2ox-3 and also by the repression of BR biosynthesis itself [58]. The repression seems to be mediated by the action of TFs such as OFP1, a member of the ovata family proteins (OFP), which interacts with DLT and targets genes of GA metabolism [59]. It was shown that, when BR concentration was elevated, overexpression of OFP1 was accompanied by reduced GA synthesis, thus assigning a role to this factor in the negative effects exerted by BR on plant cell elongation. Elevated BR signalling promotes *OFP1* gene expression and protein stability, respectively mediated by activation of OsBZR1 and inhibition of GSK2.

Similarly in Arabidopsis, it was shown that AtOFP1 (OVATE FAMILY PROTEIN1) directly targets *GA20ox-1*, a GA biosynthetic gene, to inhibit its expression [59]. Other transcription factors are involved in the inhibition of GA synthesis. One example is constituted by *OsOFP2*. Plants overexpressing *OsOFP2* showed a decreased plant height that is mediated by the regulation of *GA20ox-7* [60].

The work of Unterholzner et al. [61] also showed that in Arabidopsis, mutants lacking BR signalling have a low level of bioactive GAs and exhibit a reduction in the expression of GA biosynthetic genes of the *GA20ox* and *GA3ox* families. In addition, the application of GA to a BR signalling-deficient mutant (bri1-301) was able to rescue its developmental defects. GA application increased hypocotyl length and, at later stages, fully restored the reduced plant height and the delay in flowering time. The positive effect was detected by conducting germination experiments in which seeds were exposed in the light and directly incubated at 21 °C, without applying a cold treatment (stratification). It was thus reasoned that previous works failed to reveal this effect because the cold treatment, which is normally applied to stimulate germination, had induced GA biosynthesis [62,63,64]. The existence of a regulation at the metabolic level was also sustained by the observation that BRI1 EMS SUPPRESSOR 1 (BES1), a component of BR signalling, binds to a motif of the promoters of genes in the GA biosynthesis, such as GA20ox1 and GA3ox1, and acts as an inducer of their expression [61]. The work of Unterholzner et al. [61] indeed proposed a novel view of the regulation process mediated by GA–BR interaction, which is based on the integration between the signalling and the synthesis models. The BR signal promotes BZR1/BES1 and this leads to the increase in the GA level, thus causing the degradation of the DELLA transcriptional repressors that further releases BZR1/BES1 activity and consequently promotes plant growth.

Gao et al. [65] contributed to the dissection of BR signalling by analysing the function of the OsGAMYBL2 transcription factor and its regulatory miRNA OsmiR159. They showed that OsGAMYBL2 has a negative role on plant growth; RNAi (RNA interference) transgenic plants with reduced OsGAMYBL2 expression were taller. They also showed that OsGAMYBL2 is involved in BR signalling as well as the control of the GA pathway. This additional component in BR signalling links the canonical GSK2–BZR1 BR pathway with another downstream branch in BR signalling mediated by the transcription factor BU1 [49] and coordinates BR signalling and GA biosynthesis for the regulation of plant growth. OsGAMYBL2 negatively regulates the expression of BU1, and of CPS1 (ent-copalyl diphosphate synthase) and GA3ox2, respectively the first and last steps of GA biosynthesis [66], by binding to the promoters of these genes. The regulation of OsGAMYBL2 is quite complex and varies according to BR concentration. Without 24-epibrassinolide (eBL) treatment, OsGAMYBL2 is mainly controlled by OsmiR159d. An early response of OsMIR159d to eBL treatment was observed, in which the treatment quickly reduced the level of OsmiR159d, thus leading to an increase in the expression of OsGAMYBL2. It was also shown that OsGSK2, a key negative player in BR signalling, interacted with OsGAMYBL2 and prevented it from being degraded under 24-epibrassinolide treatment. However high level of BR caused a suppression in the activity of OsGAMYBL2 and OsGSK2 and this, in turn, eliminated the inhibitory effects on BU1 and on CPS1 and GA3ox2, thus releasing the inhibitory effect of BZR1 on other BR-related genes and promoting BR signalling and gibberellin (GA) biosynthesis. OsGAMYBL2 action is also controlled by GA. SLR1, a rice DELLA protein negatively regulating GA signalling, interacts with OsGAMYBL2 and induces its degradation, thus removing the negative action of this TF. In this way, the GA signalling enhances BR signalling [65].

Overall, the studies conducted in rice provide a complex picture of the different pathways of downstream BR responses, with different components functioning in both BR and GA pathways and coordinating the regulation of BR and GA in plant growth and development. Much less data are available in other monocot species. A study conducted in maize confirmed the view that BR and GA interaction affects specific signal transduction and responses depending on the tissue and the developmental phase. To analyse the genetic interaction between BR and GA and its role in controlling maize plant development, the work of Best et al. [20] undertook a classical genetic approach in which the authors produced F_2_ progenies segregating for either the *na1* or *na*2 mutant and a GA biosynthetic mutants, *d1* or *d5,* which are respectively defective for a 3-oxidase and the ent-kaurene synthase [67,68]. Double *na1-1/d1*, *na2-1/d1*, and *na2-1/d5* mutants were compared with single mutants. Two traits were observed whose variations are influenced by the interaction between the two hormones. Lack of GA causes the production of tillers, which does not occur in double mutants, thus showing that BR was required for the increased branching. On the other hand, GA is required for pistil production in BR mutant tassels, as tassel seed production in the BR-deficient dwarves required GA. Exogenous GA3 treatments increased the production of tassel seeds in both *na2-1* and the wild type. Different from what has been reported in rice, the study showed that BR and GA do not interact but additively affect plant height.

## 5. Conclusions and Remarks

The possibility of manipulating plant architecture is particularly challenging for plant breeders, and plant height represents one of the most interesting traits to be addressed in this context. We must consider the fact that the big transformation brought to agriculture by the green revolution was based to a great extent on the introduction of two genetic modifications, one in rice and one in wheat, which are both involved in GA metabolism and both caused a reduction of plant stature [69]. Similarly, the BR-related *uzu* mutation has successfully enhanced plant productivity in barley [34]. This latter represents an example of a selected genetic variant that has caused only a slight change in plant stature, but this change turned out to be favourable for productivity, as well as plant adaptation to the environment. With the advent of the most recent genome techniques, such as genome editing and molecular breeding, similar variants could be deliberately introduced into a number of crop species. To achieve this goal, a greater comprehension of the genetic network underlying BR metabolism and signalling is required.

As shown in Figure 1, Table 1 and Table 2, many genetic components are still lacking in the biosynthetic pathway, mostly in maize and barley. In some cases, genes have been detected in silico, but functional analysis has not been performed because corresponding mutants were not isolated. In one case involving the *DWF4* maize orthologue, the role of the encoded enzyme was characterized through a complementation test performed in Arabidopsis; however, its biological function still remains to be ascertained.

As for the signalling pathway, many components have been detected in rice, which are responsible for stimulating cell elongation and plant height (Figure 2). However, some key steps still need to be elucidated. As previously mentioned, the BR signal is perceived by OsBAK1 that phosphorylates different members of the receptor-like cytoplasmic kinase (RLCK) family. One of the members is the OsBSK3 kinase. Phosphorylation of OsBSK3 prevents binding between the TPR and kinase domains of OsBSK3, thus enabling binding between the kinase domain of OsBSK3 and a phosphatase such as bri1-SUPPRESSOR1 (BSU1). The BSU1 phosphatase [39], acting on the GSK2 kinase, has not yet been characterized in either rice or in other cereal species. Further studies should also be carried out to understand which genes and molecular regulatory mechanisms are located downstream the main TFs, such as OFP1 and DLT. It would also be important to characterize other components, besides enzymes involved in the GA synthesis, which are controlled by OsBZR1, as well as other transcription factors responding to the BR signal. In this context, the HLH transcription factors ILI1 and OsIBH1 are two interesting candidates that it would be worthy of study in further detail. These two transcription factors, as well as their Arabidopsis orthologues, are located and act downstream of BZR1 to mediate cell elongation in rice. They may also integrate different signalling pathways [48]. Other direct targets, on the basis of studies conducted in Arabidopsis [51], might be constituted by genes involved in regulation of cortical microtubule reorientation.

Rice can be considered a good model for the members of *Poaceae*. However, when knowledge is transferred to other species, one has to take into account that differences might exist. For instance, the GA–BR cross-talk is involved in controlling plant growth in rice; differently, in maize it appears not to be required for this process.

This implies that key genes might be species-specific and, consequently, breeding approaches have to be tailored to a single species.

Another important prerequisite for designing breeding strategies is the knowledge of favourable genetic variants in key genes. They could be detected by exploring the natural biodiversity existing in germplasm collections, which constitutes an important source of alleles unselected by the breeding process. The combination of favourable alleles, for more than one key gene, will be challenging for the achievement of a plant ideotype that combines positive effects on plant stature with those on other traits controlled by BR, such as an appropriate leaf angle, resistance to environmental stress, and increased seed production.

## Figures and Tables

**Table 1:** Genes involved in BR biosynthesis in rice (*Oryza sativa*), maize (*Zea mays*), and barley (*Hordeum vulgare*) described in this review, compared amongst themselves and with their correspondent genes in Arabidopsis (*Arabidopsis thaliana*).

	*Oryza sativa*		*Zea mays*		*Hordeum vulgare*		*Arabidopsis thaliana*	
Gene Product	Gene Symbol	Gene ID	Gene Symbol	Gene ID	Gene Symbol	Gene ID	Gene Symbol	Gene ID
C-24 sterol reductase	*OsBRD2*	Os10g0397400	*na2/ZmDWF1*	Zm00001d014887	*HvDIM*	KF318307	*AtDIM/AtDWF1*	AT3G19820
C3-oxidase	*OsCYP90D2/OsD2*	Os01g0197100	n.d.	n.d.	n.d.	n.d.	n.d.	n.d.
C-22 α hydroxylase	*OsCYP724B1/OsDWARF11/OsD11*	Os04g0469800	*brs1/ZmDWF4*	Zm00001d028325	n.d.	n.d.	*AtCYP90B1/AtDWF4*	AT3G50660
	*OsCYP90B2/OsDWARF4*	Os03g0227700						
5α Reductase	*OsDET2*	Os01g0851600	*na1/Zmdet2*	Zm00001d042843	n.d.	n.d.	*AtDET2/AtDWF6*	AT2G38050
		Os11g0184100						
C-23α-hydroxylase/C-3 dehydrogenase	*OsCYP90A3/OsCPD1*	Os11g0143200	n.d.	n.d.	*CYP90A1/HvCPD*	KF360233	*AtCYP90A1/AtCPD/AtDWF3*	AT5G05690
	*OsCYP90A4/OsCPD2*	Os12g0139300						
C-23 hydroxylases	n.d.	n.d.	n.d.	n.d.	n.d.	n.d.	*AtCYP90C1/AtROT3*	AT4G36380
							*AtCYP90D1*	AT3G13730
Brassinosteroid-6-oxidase 1	*OsCYP85A1/OsDWARF/OsBRD1*	Os03g0602300	*lil1/Zmbrd1*	Zm00001d033180	*HvBRD*	KF318308	*AtCYP85A1/AtBR6ox1*	AT5G38970
Brassinosteroid-6-oxidase 2	n.d.	n.d.	n.d.	n.d.	n.d.	n.d.	*AtCYP85A2/AtBR6ox2*	AT3G30180

Gene symbol and models were retrieved from https://rapdb.dna.affrc.go.jp/index.html (RAP-DB), https://www.maizegdb.org/gbrowse/maize_v4 (MaizeGDB), https://www.ncbi.nlm.nih.gov/genbank/ (GenBank/EMBL), and https://www.arabidopsis.org/index.jsp (TAIR) for rice, maize, barley, and Arabidopsis respectively.

**Table 2:** Phenotypic alteration occurring in BR biosynthesis mutants of rice (*Oryza sativa*), maize (*Zea mays*), and barley (*Hordeum vulgare*) described in this review.

	*Oryza sativa*			*Zea mays*			*Hordeum vulgare*		
Gene Product	Gene Symbol	Mutant Allele	Mutant Phenotype	Gene Symbol	Mutant Allele	Mutant Phenotype	Gene Symbol	Mutant Allele	Mutant Phenotype
C-24 sterol reductase	*OsBRD2*	*brd2*	Moderate dwarf seedlings and severe dwarf adult plant, defective root elongation, dark-green and erect leaves, shortened leaf sheaths, malformed panicles and shorter grains.	*na2/ZmDWF1*	*na2*	Extreme dwarf, feminized tassels, reduced branching, upright leaves.	*HvDIM*	*ari-o; brh; brh14; brh16; ert-u; ert-zd*	Semidwarf, breviaristatum, brachytic, short culm, erect and upright leaves.
C3-oxidase	*OsD2*	*d2*	Mild semidwarf, erect leaves, shorter grains.	n.d.	n.d.	n.d.	n.d.	n.d.	n.d.
C-22 α hydroxylase	*OsD11*	*d11*	Semidwarf, erect leaves, shortening of the second internode in culm, reduced grain length.	*brs1/ZmDWF4*	n.d.	In Arabidopsis, constitutive expression of ZmDWF4 complements DWF4 mutants.	n.d.	n.d.	n.d.
	*OsDWARF4*	*dwarf4*	Slightly dwarfed stature, erect leaves without abnormal leaf, flower and grain morphology.						
5α Reductase	*OsDET2*	*n.d.*	n.d.	*na1/Zmdet2*	*na1*	Dwarf, reduction of internode length, erect leaves, feminizes male flowers.	n.d.	n.d.	n.d.
C-23α-hydroxylase/C-3 dehydrogenase	*OsCPD1*	*oscpd1*	No BR-deficient phenotype.	n.d.	n.d.	n.d.	*HvCPD*	*brh13; brh18*	Semidwarf, brachytic, short culm, erect and upright growth.
	*OsCPD2*	*n.d.*	n.d.						
C-23 hydroxylases	n.d.	*n.d.*	n.d.	n.d.	n.d.	n.d.	n.d.	n.d.	n.d.
Brassinosteroid-6-oxidase 1	*OsBRD1*	*brd1*	Extreme dwarfism, completely defective in internode elongation, short leaf sheaths, short curled and frizzled leaf blades, defective root elongation, no panicles or rarely small and sterile seeds.	*lil1/Zmbrd1*	*brd1; lil1*	Severe dwarfism, feminized tassels, reduced branching, upright leaves.	*HvBRD*	*ari-u; brh3; ert-t*	Semidwarf, breviaristatum, brachytic, short culm, erect and upright leaves.
**Brassinosteroid-6-oxidase 2**	n.d.	*n.d.*	n.d.	n.d.	n.d.	n.d.	n.d.	n.d.	n.d.
